# QEEG Measures in Huntington's Disease: A Pilot Study

**DOI:** 10.1371/currents.RRN1192

**Published:** 2010-10-25

**Authors:** Aimee Hunter, Yvette Bordelon, Ian Cook, Andrew Leuchter

**Affiliations:** ^*^UCLA School of Medicine; ^†^Department of Neurology UCLA; ^‡^UCLA Semel Institute and ^§^UCLA

## Abstract

Structural brain changes as measured with Magnetic Resonance Imaging (MRI) are associated with progression of Huntington’s Disease (HD), a trinucleotide repeat neurodegenerative disorder. Neurophysiological measures may offer additional biomarkers of the onset and progression of brain disease. We used quantitative electroencephalography (QEEG) power measures to assess resting state brain function in 27 HD subjects and 15 healthy controls. Those QEEG features that distinguished between HD subjects and healthy controls were examined in relation to illness severity, using Unified Huntington Disease Rating Scale (UHDRS) subscales, as well as to the number of CAG repeats in the HD cohort. HD subjects showed a global increase in delta power as compared to controls, even when examining unmedicated HD subjects only (n = 13), or premanifest HD subjects only (n = 3). HD subjects also showed loss of the normal anterior-posterior (AP) gradient of relative alpha and delta power. Relative alpha AP gradient loss was associated with lower Total Functional Capacity (TFC) and greater cognitive dysfunction. Relative delta AP gradient loss was associated with lower TFC, more severe motor symptoms, and greater number of CAG repeats. Overall, results suggest that QEEG power measures may capture perturbations of brain function that are related to functional status as well as to underlying genetic repeat expansion in HD. Pilot data in the three premanifest HD subjects are consistent with the hypothesis that brain functional abnormalities may be detectable even in premanifest gene carriers. Cross-sectional findings suggest that QEEG measures may be biomarkers of HD progression; prospective studies in larger samples are needed to confirm these findings and test hypotheses regarding underlying mechanisms.

**Introduction**

Huntington’s Disease (HD) is a progressive neurodegenerative disorder that affects approximately 30,000 persons in the US.  There is no known treatment that alters the course of the disease.  HD is caused by an enlarged CAG repeat expansion in the huntingtin protein gene on the short arm of chromosome 4 that is characterized by a triad of symptoms:  hyperkinetic movement disorder, cognitive dysfunction, and behavioral changes[1].  The hallmark of HD pathology is striatal atrophy, the severity of which is the basis for the Vonsattel grading system[2].  Imaging studies have documented that this striatal volume can be detected years before the onset of motor symptoms[3] [4].  Cortical atrophy is also found in HD both in symptomatic and premanifest subjects with progressive cortical thinning detectable in a posterior to anterior direction with increasing disease severity, the largest effects being observed in the sensorimotor cortex [5] [6] [7].

A biomarker that captured subtle brain dysfunction in the premanifest stages and early phases of illness, and that served as a surrogate marker for illness progression, could facilitate the development of treatments to alter the course of HD.  Some functional brain imaging studies have found that striatal and cortical brain glucose metabolism as measured by fluorodeoxyglucose (FDG) positron emission tomography (PET) is altered in both premanifest and symptomatic HD [8] [9] [10] [11] [12].  In this study, we aimed to investigate whether quantitative electroencephalography (QEEG) could form the basis for such a biomarker.  QEEG has the advantage of being affordable, easily obtainable, and non-invasive[13], and previous studies have documented EEG abnormalities in HD[14].  Decreases in absolute alpha power have been reported in symptomatic HD [15] [16] [17] [18] [19] [20] as well as in premanifest gene carriers [17].  Although deTommaso and colleagues did not find correlations between cognitive dysfunction and alterations in absolute alpha power in HD [17], one additional study of premanifest HD subjects found decreases in alpha power during a working memory task [21].  Increases in beta and delta power also have been reported in HD [16].

To further explore the utility of QEEG as a potential biomarker for HD, we examined QEEG power measures in premanifest, mild, moderate, and severe HD subjects as well as in healthy controls.  We compared QEEG power measures between HD and control subjects, and assessed the relationship between QEEG power measures and level of illness severity in HD, to determine whether these measures could detect brain functional differences associated with different stages of illness.

**Methods**

**Subjects and Clinical Assessments:** 27 HD subjects and 15 healthy controls enrolled through the Huntington Disease Clinic (HDC) and the Laboratory of Brain, Behavior, and Pharmacology at UCLA.  All subjects provided written informed consent in accordance with the Declaration of Helsinki prior to participation in IRB-approved procedures.  Historical and demographic information was collected including gender, age, disease duration, and current medications. CAG repeat length was collected from prior HDC genetic evaluations.  The Unified Huntington Disease Rating Scale (UHDRS) was used to measure motor, cognitive, behavioral, and general function in the HD cohort.  Subjects were stratified according to disease severity as previously established [22] based on Total Functional Capacity (TFC) score of the UHDRS.  Categories included mild (TFC of 11-13), moderate (TFC of 7-10), severe (TFC of 0-6), or premanifest (no or nonspecific motor symptoms with a UHDRS total motor score (TMS) < 5).  TFC measures impairment in daily tasks including work, management of household finances and chores, and self-care with a high score of 13 indicating normal function and diminished capabilities recognized by lower scores.  The TMS assesses eye movement abnormalities, chorea, dystonia, bradykinesia, rigidity, gait and balance and ranges from 0 (no motor symptoms) to a maximum of 124 (most severe).  UHDRS cognitive tests assess executive functions, primarily frontal-subcortical tasks, and include verbal fluency using the Controlled Oral Word Association Task, Digit-Symbol Substitution Task, and the Stroop Test.  The Folstein modified Mini-Mental State Examination (MMSE; [23]) also was administered.

**QEEG Procedures:**  QEEG recordings were obtained from subjects in the awake resting state in a manner similar to that employed clinically.  Electrodes were placed using a standard extension of the International 10-20 system (Figure 1).  Data were recorded in real-time using a bandpass filter of 0.3 to 70 Hz, and were digitized at a rate of 250 samples/channel/second with a Pz reference as previously described[24].  Two-second epochs of data for quantitative analysis were selected by qualified technicians according to standard procedures using the first 20-32 seconds of artifact-free EEG.  Data were processed offline to obtain linked-ears absolute and relative power in four frequency bands (0.5-4 Hz, 4-8 Hz, 8-12 Hz, and 12-20 Hz).  Power was calculated using Brain Vision Analyzer software (Brain Product GmbH; Gilching, Germany).  Global power in each band was calculated by averaging power values for all 35 channels.  Anterior-posterior (AP) power gradients were calculated for relative alpha and delta power bands according to Cook and colleagues [25] where: AP gradient = ((anterior-posterior)/(anterior + posterior)). Anterior electrodes included: Fp1, F3, F7, Fp2, F4, F8, Fz, and Cz; posterior electrodes included: P3, O1 P4, O2 and Pz (Figure 1).

**Figure:**
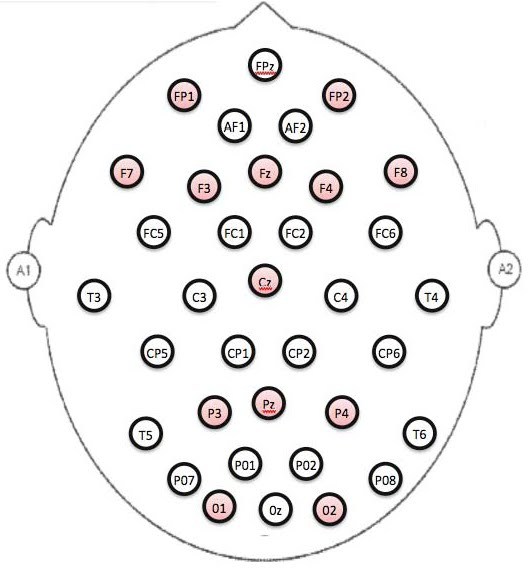
**Figure 1.** Electrodes placement using a standard extension of the International 10-20 system. Electrodes highlighted in red were used to calculate anterior-posterior (AP) gradients.

**Statistical Analysis: **Wilcoxon–Mann–Whitney tests were used to assess differences in QEEG power measures between HD subjects and healthy controls.  We compared all HD subjects to controls, and also examined subsets of the HD group (medication-free HD subjects, premanifest HD subjects) as compared to controls to further explore the utility of these biomarkers.  Spearman bivariate correlations were conducted to evaluate relationships between QEEG power measures and measures of HD illness severity including TFC, number of CAG repeats, and measures of cognitive and motor function.**  **Significance level was set at p ≤ .05.  Because this was an exploratory study, we reported actual p-values without correction for multiple comparisons.

**Results**

**Baseline Demographic and Clinical Characteristics**

HD subjects (n = 27) had a mean age of 46.4 (±11.3) years with F:M ratio of 11:16, and controls (n = 15) had a mean age of 47.9 (±9.7) years with F:M ratio of 8:7.  Illness severity in the HD subjects was distributed as follows:  premanifest (n = 3), mild (n=15), moderate (n=6), and severe (n=3) HD. Clinical and demographic characteristics of HD subjects are shown in Table 1.  Consistent with greater disease severity, HD subjects with lower TFC scores exhibited numerically higher Total Motor Symptom (TMS) scores, and greater impairment on cognitive tasks.  For the three premanifest subjects in our sample, we estimated years to onset calculated according to the method of Langbehn and colleagues [26].  Fifty-two percent of HD subjects were taking one or more concomitant CNS active medications that could influence the EEG.  Concurrent medications included: buspirone, gabapentin, mirtazepine, quetiapine, trazodone, olanzapine, alprazolam, bupropion, escitalopram, memantine, paroxetine, sertraline, and zolpidem.  Therefore, in addition to comparing all HD subjects to healthy controls, we also conducted analyses using only the medication-free subset (n = 13) of HD subjects.

**Table 1.  Clinical and Demographic Characteristics of HD subjects.**

**Table:** 

	**Premanifest** **(n=3) **	**Mild** **(n=15)**	**Moderate** **(n=6)**	**Severe** **(n=3)**	**All HD** **(n=27)**
**Age (yrs)**	32.7 (2.5)	49.4 (9.9)	46.7 (14.6)	45.0 (8.9)	46.4 (11.3)
**Gender (F:M)**	2:1	2:3	1:1	0:3	11:16
**Duration (yrs)**	14 (4) to onset	5.3 (4.2)	7.3 (3.1)	14.0 (7.2)	6.9 (5.1)
**CAG repeat length**	43.67 (1.16)	43.87 (3.18)	46.60 (5.03)	46.67 (4.04)	44.69 (3.59)
**Concomitant** **Medication**	0	53%	67%	67%	52%
**TFC**	13 (0)	11.9 (0.7)	8.0 (1.3)	5.3 (1.2)	10.4 (2.7)
**TMS**	0.3 (0.6)	27 (7.8)	55.3 (13.7)	40.0 (13.7)	31.8 (18.6)
**MMSE**	29.7 (.58)	27.9 (2.2)	25.5 (5.8)	25.5 (5.0)	27.3 (3.6)
**Verbal Fluency**	49.7 (21.0)	33.2 (12.7)	18.3 (9.2)	20.3 (8.5)	30.2 (15.4)
**Digit symbol**	51.0 (10.1)	29.3 (10.1)	22.3 (8.5)	19.3 (2.9)	29.0 (12.5)
**Stroop Color**	71.3 (22.7)	49.9 (16.1)	38.3 (11.1)	28.0(5.7)	47.9 (18.4)
**Stroop Word**	86.0 (15.1)	67.7 (20.5)	50.3 (13.3)	35.0 (7.1)	63.1 (21.7)
**Stroop Interference**	40.0 (14.0)	31.4 (7.8)	21.5 (7.0)	17.0 (5.7)	28.9 (10.2)

Abbreviations: TFC=Total Functional Capacity of the UHDRS; TMS=Total Motor Score of the UHDRS, MMSE=Mini Mental State Exam; Values are means with standard deviations in parentheses.

**QEEG measures in HD subjects versus Controls**

Whole-brain topographic maps were generated for HD subjects and controls in each of four frequency bands (delta, 0.5-4 Hz; theta, 4-8 Hz; alpha, 8-12 Hz; beta, 12-20 Hz).  Group map comparisons between HD and control subjects revealed visually salient differences in the alpha and delta frequency bands (Figure 2).  Statistical analyses therefore were performed to examine these measures.  Table 2 shows mean differences in the various power measures between healthy controls and HD groups including: (a) all HD subjects, (b) unmedicated HD subjects, and (c) premanifest HD subjects.   

*Global QEEG measures*

HD subjects showed a trend toward greater global (i.e., whole head average) absolute alpha power as compared to controls (Figure 2A);  however, the trend did not persist when medication-free HD subjects were compared to controls (Table 2).  In contrast, the absolute *delta* power measure differed consistently between controls and each HD comparison group (all HD, unmedicated HD, and premanifest HD) (Figure 2B; Table 2).Topographic maps of relative alpha or delta power (Figures 2C and D) did not show any apparent global differences between HD subjects and healthy controls and therefore no statistical tests were performed.

*Anterior-posterior gradients*

Inspection of the maps of relative power (Figures 2C and D) revealed brain regional differences in the distribution of relative alpha and delta power between healthy subjects and those with HD.  Healthy subjects showed an anterior-posterior (AP) gradient spanning from low relative alpha values anteriorly to high values posteriorly, with the reciprocal pattern in the delta band.  In contrast to the healthy subjects, HD subjects showed an apparent loss of this anterior-posterior relative power gradient both in relative alpha and relative delta frequencies.  Statistical results of group comparisons are shown in Table 2.  Using a quantified measure of the anterior-posterior (AP) gradient [27], both the relative alpha power AP gradient and the relative delta power AP gradient were significantly different between HD subjects and healthy controls, even when considering only medication-free HD subjects. In addition, the relative alpha AP gradient distinguished between the three premanifest HD subjects and healthy controls (p =.04). 

**Table 2. QEEG power measures: Comparisons between HD groups and controls.**

**Table:** 

	** ** **Controls** **(n=15)**	**(a)** **All HD** **(n=27)**	** ** ** ** ** ** **Sig. test**	** ** **(b)** **Unmedicated** **HD (n=13)**	** ** ** ** ** ** ** ** **Sig. test**	** ** **(c)** **Premanifest** **HD (n=3)**	** ** ** ** ** ** **Sig. test**
**Global absolute** **alpha**	18.12 (15.04)	56.09 (62.61)	z = -1.719; p = .086	52.24 (74.21)	z = -.990; p = .332	33.87 (41.55)	z = -.296; p = .767
**Global absolute** **delta**	10.11 (3.01)	43.45 (10.83)	z = -5.316**; p = .0000001	41.08 (8.32)	z = -4.491**; p = .000007	43.27 (1.45)	z = -2.666**; p = .008
**Relative** **alpha AP gradient**	-0.40 (0.13)	-0.10 (0.11)	z = -4.633**; p = .000004	-0.12 (0.12)	z = -3.893**; p = .0001	-0.18 (0.11)	-2.073*; p = .038
**Relative** **delta AP gradient**	0.37 (0.23)	0.12 (0.13)	z = -3.137**; p = . 002	0.13 (0.14)	z = -2.511*; p = . 012	0.10 (0.06)	z = -1.599 ; p = .110

 Mean QEEG power (± standard deviation) in healthy controls as compared to (a) all HD subjects, (b) medication-free HD subjects, and (c) premanifest HD subjects.   *p ≤ 0.05; ** p ≤ 0.01

**Figure:**
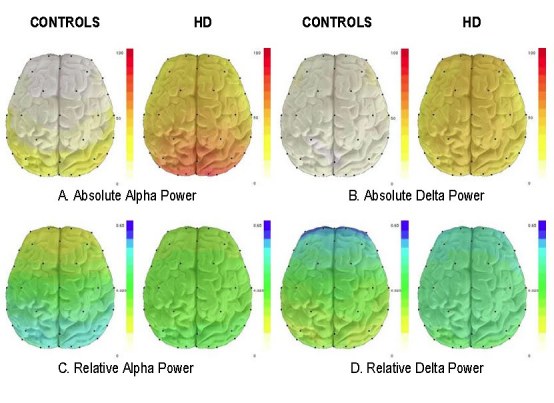
**Figure 2.** Alpha and delta absolute and relative power in healthy controls (n=15) and HD subjects (n=27). Warmer colors represent higher absolute power; blue represents higher relative power.

**QEEG measures, HD severity and other Clinical Measures**

Each of the four QEEG power measures that distinguished between HD and controls (i.e., global absolute alpha, global absolute delta, relative alpha AP gradient, relative delta AP gradient) was examined in relation to TFC score, CAG repeat length, and the complement of clinical and functional measures evaluated in our HD sample (Table 3).  In addition to examining CAG repeat length, we calculated a burden of pathology score (BPS) for each subject based on the work of Penney and colleagues [28].  This measure (BPS = [CAG-35.5] x age) serves as an index of disease burden that accounts for subject age (cf. [7]).   

**Table 3. ** **Spearman correlations between QEEG and illness severity measures**

**Table:** 

**Subject Variable**	**Global absolute alpha**	**Global absolute delta**	**Relative alpha ** **AP gradient**	**Relative delta ** **AP gradient**
CAG repeats (n=26)	-.152 p = .460	-.084 p = 0.683	.055 p = 0.790	-.507** p = 0.008
DBS (n=26)	.003 p = .988	-.103 p = .618	.327 p = .103	-.431* p = .028
TFC (n=27)	-.219 p = .272	.244 p = .221	.561** p = .002	.391* p = .044
TMS (n=27)	-.078 p = .699	-.290 p = .143	.436* p = .023	-.390* p = .044
MMSE (n=24)	-.508* p = .011	-.069 p = .750	-.382 p = .066	.062 p = .772
Verbal Fluency (n=26)	-.220 p = .280	.246 p = .227	-.341 p = .088	-.072 p = .727
Digit Symbol (n=26)	-.402* p = .042	.004 p = .985	-.386 p = .052	-.037 p = .858
Stroop Color (n=25)	-.199 p = .340	-.039 p = .852	-.402* p = .047	.201 p = .336
Stroop Reading (n=25)	-.411* p = .041	-.204 p = .328	-.592** p = .002	.166 p = .427
Stroop Interference (n=25)	-.300 p = .145	.113 p = .592	-.488* p = .013	.057 p = .788

Abbreviations: DBS=Disease Burden Score; TFC=Total Functional Capacity of the UHDRS; TMS=Total Motor Score of the UHDRS, MMSE=Mini Mental State Exam. *p ≤ 0.05; ** p ≤ 0.01

Global absolute delta did not correlate significantly with CAG repeats or with any functional measure.  Global absolute alpha was inversely correlated with the MMSE, Digit Symbol, and Stroop reading.  Loss of the AP gradient in relative alpha and relative delta bands was associated with lower functional capacity and poorer cognitive performance on specific measures.  Relative delta AP gradient showed a significant inverse correlation with number of CAG repeats, BPS, and Total Motor Score (TMS), as well as a positive association with TFC.  Relative alpha AP gradient was positively correlated with BPS, and inversely correlated with TFC (Figure 3) and all three Stroop components (Table 3).   Figure 4 shows mean relative alpha AP gradient values for healthy control subjects and for HD subjects grouped according to illness severity category.

**Figure:**
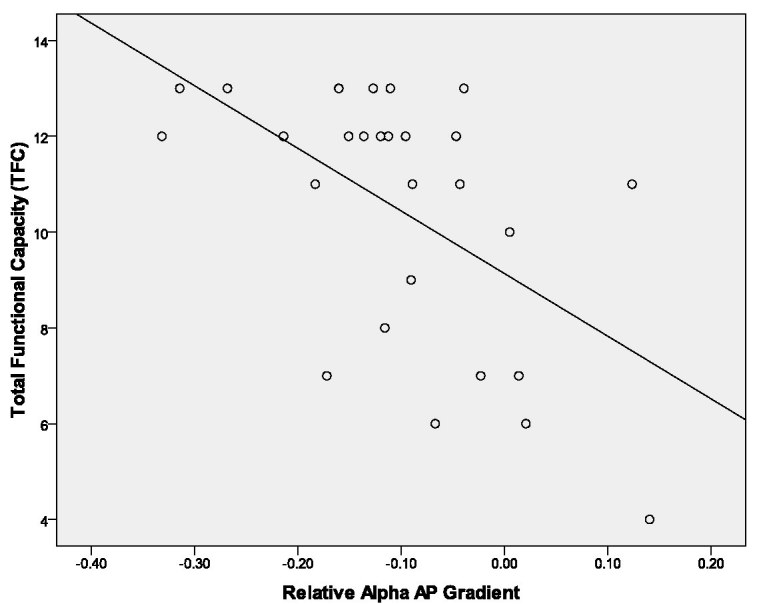
**Figure 3.** Relationship between relative alpha AP gradient and TFC scores in HD subjects (n=27).

**Figure:**
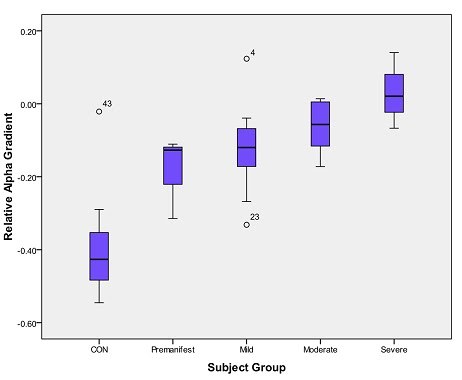
**Figure 4.** Relative alpha AP gradient values for healthy controls and HD subjects in the various illness severity groups. Values are expressed as means with the box representing the interquartile range and line indicating the standard deviation.

In order to examine the earliest stages of HD, we further explored the relative alpha AP gradient in the three premanifest HD subjects.  ‘Estimated times to onset’ [26] for each of the three premanifest subjects were 18, 14, and 10 years, respectively.  Although the number of subjects was very small, the relative alpha AP gradient which was associated with illness severity in manifest HD also appeared to be associated with shorter time to onset (*r*_*s*_  =1.00) (Figure 5).

**Figure:**
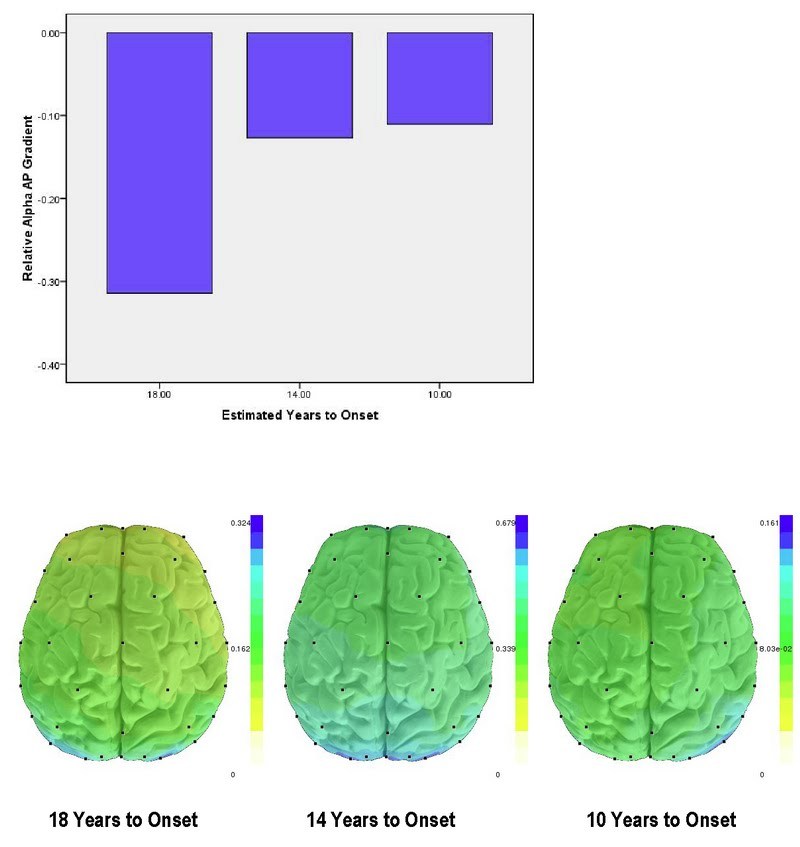
**Figure 5.** Relative alpha AP gradient in the three premanifest HD subjects. Estimated years to onset of symptomatic disease were determined by the Langbehn method. These three cases suggest a shift in the relative alpha gradient, from posterior prominence to loss of the gradient with approaching HD onset. Brain illustrations show higher relative alpha values in blue, lower values in yellow.

**Discussion**

QEEG power measures appeared to detect both global and regional brain dysfunction in HD.  Several measures may have especially strong promise as candidate biomarkers of HD and HD severity.  HD subjects showed higher levels of global absolute delta power as compared to healthy controls, and this difference also was seen in a small number of asymptomatic HD gene carriers.  Higher absolute delta in the HD group did not appear to be due to concomitant medication use, as we found a significant difference also between unmedicated HD subjects and controls.  This result is consistent with previous reports of increased delta power in HD [16] [17].  Increases in delta power are seen in neurodegenerative diseases in general, and are interpreted as being indicative of cortical disease [29].  It is known that metabolic dysfunction is part of the pathophysiological cascade leading to neuronal death in HD [30].  QEEG measures of power therefore may reflect underlying metabolic stress occurring as part of HD.  Indeed, functional imaging studies have demonstrated decreased basal metabolism in frontal and temporal cortices in symptomatic and premanifest disease [9] [14] [31] [32].  Our finding that global absolute delta power was altered in even premanifest gene carriers suggests that this measure may track with worsening cortical disease independent of manifest symptoms, although only a small number of premanifest subjects were examined in this study.  Global absolute delta did not correlate with any illness severity measures in our HD sample.

Of note, there was a trend toward higher global absolute alpha power in our HD subjects when compared to controls.  This result is in contrast to previous EEG studies in HD which demonstrated decreases in alpha power [15] [16] [17].  Differences between our results and those of previous reports may reflect differences in age, medication, or general medical condition between subjects in the current study and those of previous studies.  Most previous studies did not report the details of psychotropic medication use in their subjects.  In our sample, the trend was no longer evident when comparing medication-free or premanifest HD subjects to controls.

Regional patterns of relative alpha power and relative delta power showed robust differences between HD and controls.  Regarding relative alpha, whereas healthy subjects exhibited greater relative alpha power in posterior versus anterior regions (i.e., a negative AP gradient), the HD subjects showed a loss of this AP gradient.  Relative alpha AP gradient showed strong discrimination between HD and healthy controls even when considering only those HD subjects who were medication free.  This measure also distinguished premanifest HD subjects from healthy controls.  Importantly, relative alpha AP gradient also appeared to have sensitivity to the degree of illness severity.  Relative alpha AP gradient showed a strong (i.e.,* r*_s_ = -.56) correlation with TFC score, one of the most widely used measures of disease progression [33], and was associated with cognitive functional measures including color, reading, and interference components of the Stroop, and a trend (p = .052) association with Digit Symbol.  Intriguingly, consistent with the direction of the finding in the total HD sample, examination of the three premanifest subjects showed a numeric association between loss of the relative alpha AP gradient and shorter estimated time to onset [26].  Brain regional differences in relative alpha AP gradient between HD and controls were driven primarily by a decrease in posterior relative alpha power in HD.  Structural imaging of HD subjects has shown more predominant posterior cortical thinning [6] [7]; loss of the relative alpha power gradient in our sample may reflect this anatomical finding.

Regarding the AP gradient of relative delta power, whereas controls showed greater relative delta in the anterior regions (i.e., a positive gradient), HD subjects showed a loss of this AP gradient.  This measure was significantly different in HD and medication-free HD subjects as compared to controls, but was not significantly different in premanifest HD subjects.  Relative delta power gradient correlated with both TFC and TMS.   Loss of the relative delta AP gradient also was found to correlate with CAG repeat length and the related BPS measure which accounts for both CAG repeats and subject age [28].  CAG repeat length is an especially important marker in HD because of its association with earlier onset and faster progression of disease, although there is great variability among patients with the same CAG repeat length [28] [34] [35] [36].  Relative delta AP gradient was the only power measure of those that we analyzed that correlated with CAG repeat length.

Results of this hypothesis-generating study should be considered within the following limitations.  First, findings should be interpreted cautiously due to the small number of subjects with a wide range of illness severity, including a premanifest HD subgroup which consisted of only three subjects.  Future work should expand the sample size particularly in the premanifest cohort which is of special interest for detecting early brain functional changes in the EEG.  Second, the cross-sectional design does not allow for a direct assessment of how biomarkers may change with changing illness severity.  Longitudinal studies are required to determine whether EEG changes actually track with illness progression across time.  Third, EEG measurements were obtained with some subjects concomitantly taking medications known to influence the EEG.  Although we conducted separate analyses of unmedicated HD subjects to determine whether differences between HD and control subjects were due to medication effects, analyses of the pooled sample (medicated and unmedicated subjects) include medication effects as a source of uncontrolled variance.  Fourth, we assessed illness severity using the TFC, an index that may have limited sensitivity for progression of HD in certain stages of disease [37].   Recent work, however, has shown that the TFC captures the earliest functional declines among subjects who phenoconvert to manifest HD [38].  Future studies should incorporate more sensitive measures as they become validated.

Overall findings of this exploratory study find support for QEEG as a biomarker of HD.  Several measures showed excellent discrimination between HD and controls, and appeared to separate even premanifest HD subjects from controls. AP gradient measures appeared to capture not only differences between HD and controls, but also differences in HD illness severity.  Although our study was cross-sectional, these findings suggest that QEEG power measures may track with illness progression, even in the earliest stages of illness.  Future prospective, longitudinal studies should replicate these exploratory findings and assess whether these QEEG power measures change over time with advancing illness.  Confirmation of these findings could yield surrogate markers to aid in developing treatments to halt or slow progression of the illness.****

**Acknowledgements**

The authors thank Barbara Siegman, M.A., R.EEG.T. for EEG recording and processing.

**Correspondence** should be directed to Aimee M. Hunter, Ph.D.: Semel Institute for Neuroscience and Human Behavior at UCLA; 760 Westwood Plaza, Rm. 37-359; Los Angeles, CA  90024-1759; Phone: (310) 206-2237; FAX: (310) 825-7642; e-mail: amhunter@ucla.edu .

**Funding Information**

Funding for this study was provided by CHDI Foundation, Inc.   CHDI had no role involvement in the conduct of the research and/or preparation of the article; in the collection, analysis and interpretation of data; in the writing of the report; and in the decision to submit the paper for publication.

**Competing Interests**

Dr. Hunter has been funded solely through NIH grants and institutional support. 

Dr .Bordelon has received grant funding from the National Institute of Nuerological Disorders and Stroke (NINDS) and the Michael J. Fox Foundation, and has received Honoraria from Lundbeck Inc. and Teva Neurosciences. 

Dr. Cook has received grant support from Aspect Medical Systems, Cyberonics, Eli Lilly and Company, the John A. Hartford Foundation, MedAvante, the National Institutes of Health, Neuronetics, Novartis, Pfizer, Vivometrics, and the West Coast College of Biological Psychiatry; has served as a consultant to Ascend Media, Bristol-Myers Squibb, Cyberonics, Eli Lilly and Company, Forest Laboratories, Janssen, Neuronetics, Scale Venture Partners, and the U.S. Department of Justice; and has been a member of the speakers' bureau for Bristol-Myers Squibb, CME LLC, Medical Education Speakers Network, Pfizer, and Wyeth. Dr. Cook is not a shareholder in any pharmaceutical or medical device company; his patents are assigned to the University of California. 

Dr. Leuchter has provided scientific consultation or served on advisory boards for Aspect Medical Systems, Bristol-Myers Squibb, Eli Lilly and Company, Merck & Co., Otsuka Pharmaceuticals, and Pfizer.  He has served on a speaker's bureau for Bristol-Myers Squibb, Eli Lilly and Company, Otsuka Pharmaceuticals, and Wyeth-Ayerst Pharmaceuticals.  He has received research/grant support from the National Institute of Mental Health, the National Center for Complementary and Alternative Medicine, Aspect Medical Systems, Eli Lilly and Company, Wyeth-Ayerst Pharmaceuticals, Merck & Co., Pfizer, Sepracor, Vivometrics, and MedAvante.  

## References

[ref-890202660] Ho, A.K., Sahakian, B.J., Brown, R.G., Barker, R.A., Hodges, J.R., Ané, M.N., Snowden, J., Thompson, J., Esmonde, T., Gentry, R., Moore, J.W., Bodner, T.; NEST-HD Consortium, 2003. Profile of cognitive progression in early Huntington's disease. Neurology. 61, 1702-1706. 10.1212/01.wnl.0000098878.47789.bd 14694033

[ref-484913247] Vonsattel, J. P., Myers, R. H., Stevens, T. J., Ferrante, R. J., Bird, E. D., and Richardson, E. P., Jr., 1985. Neuropathological classification of Huntington's disease. J Neuropathol Exp Neurol 44, 559-577. 10.1097/00005072-198511000-00003 2932539

[ref-3227596945] Aylward, E.H., Sparks, B.F., Field, K.M., Yallapragada, V., Shpritz, B.D., Rosenblatt, A., Brandt, J., Gourley, L.M., Liang, K., Zhou, H., Margolis, R.L., Ross, C.A., 2004. Onset and rate of striatal atrophy in preclinical Huntington disease. Neurology. 63, 66-72. 10.1212/01.wnl.0000132965.14653.d1 15249612

[ref-3780901142] Paulsen, J S, Langbehn, D R, Stout, J C, et al. 2008 . Detection of Huntington's disease decades before diagnosis: the Predict-HD study. Journal of Neurology, Neurosurgery and Psychiatry, 79, 874-80. 10.1136/jnnp.2007.128728 PMC2569211 18096682

[ref-181981819] Rosas, H.D., Liu, A.K., Hersch, S., Glessner, M., Ferrante, R.J., Salat, D.H., van der Kouwe, A., Jenkins, B.G., Dale, A.M., Fischl, B., 2002. Regional and progressive thinning of the cortical ribbon in Huntington's disease. Neurology. 58, 695-701. 10.1212/wnl.58.5.695 11889230

[ref-1907911278] Rosas, H.D., Salat, D.H., Lee, S.Y., Zaleta, A.K., Pappu, V., Fischl, B., Greve, D., Hevelone, N., Hersch, S.M., 2008. Cerebral cortex and the clinical expression of Huntington's disease: complexity and heterogeneity. Brain. 131, 1057-1068. 10.1093/brain/awn025 PMC2657201 18337273

[ref-714495934] Tabrizi, S.J., Langbehn, D.R., Leavitt, B.R., Roos, R.A., Durr, A., Craufurd, D., Kennard, C., Hicks, S.L., Fox, N.C., Scahill, R.I., Borowsky, B., Tobin, A.J., Rosas, H.D., Johnson, H., Reilmann, R., Landwehrmeyer, B., Stout, J.C.; TRACK-HD investigators, 2009. Biological and clinical manifestations of Huntington's disease in the longitudinal TRACK-HD study: cross-sectional analysis of baseline data. Lancet Neurol. 8(9), 791-801. 10.1016/S1474-4422(09)70170-X PMC3725974 19646924

[ref-397170187] Antonini, A., Leenders, K. L., Spiegel, R., Meier, D., Vontobel, P., Weigell-Weber, M., Sanchez-Pernaute, R., de Yebenez, J. G., Boesiger, P., Weindl, A., and Maguire, R. P., 1996. Striatal glucose metabolism and dopamine D2 receptor binding in asymptomatic gene carriers and patients with Huntington's disease. Brain 119 ( Pt 6), 2085-2095. 10.1093/brain/119.6.2085 9010012

[ref-2375157506] Ciarmiello, A., Cannella, M., Lastoria, S., Simonelli, M., Frati, L., Rubinsztein, D.C., Squitieri, F., 2006. Brain white-matter volume loss and glucose hypometabolism precede the clinical symptoms of Huntington's disease. J Nucl Med. 47, 215-222. 16455626

[ref-1044157728] Feigin, A., Leenders, K. L., Moeller, J. R., Missimer, J., Kuenig, G., Spetsieris, P., Antonini, A., and Eidelberg, D., 2001. Metabolic network abnormalities in early Huntington's disease: an [(18)F]FDG PET study. J Nucl Med 42, 1591-1595. 11696626

[ref-2524490356] Kuhl, D. E., Phelps, M. E., Markham, C. H., Metter, E. J., Riege, W. H., and Winter, J., 1982. Cerebral metabolism and atrophy in Huntington's disease determined by 18FDG and computed tomographic scan. Ann Neurol 12, 425-434. 10.1002/ana.410120504 6217782

[ref-1515401960] Kuwert, T., Lange, H. W., Langen, K. J., Herzog, H., Aulich, A., and Feinendegen, L. E., 1990. Cortical and subcortical glucose consumption measured by PET in patients with Huntington's disease. Brain 113 ( Pt 5), 1405-1423. 10.1093/brain/113.5.1405 2147116

[ref-283908303] Paulsen, J.S., 2009. Biomarkers to predict and track diseases. Lancet Neurol. 8(9), 776-7. 10.1016/S1474-4422(09)70203-0 PMC3797569 19646925

[ref-3821020417] Nguyen, L., Bradshaw, J.L., Stout, J.C., Croft, R., Georgiou-Karistianis, N., 2010. Electrophysiologicalmeasures as potential biomarkers in Huntington’s disease: Review and future directions. Brain Research Reviews. 10, 10-16. 10.1016/j.brainresrev.2010.03.004 20381528

[ref-1934334636] Bellotti, R., De Carlo, F., Massafra, R., de Tommaso, M., Sciruicchio, V., 2004. Topographic classification of EEG patterns in Huntington's disease. Neurol Clin Neurophysiol. 2004, 37. 16012598

[ref-4198277341] Bylsma, F.W., Peyser, C.E., Folstein, S.E., Folstein, M.F., Ross, C., Brandt, J., 1994. EEG power spectra in Huntington's disease: clinical and neuropsychological correlates. Neuropsychologia. 32, 137-150. 10.1016/0028-3932(94)90001-9 8190239

[ref-2745545021] de Tommaso, M., De Carlo, F., Difruscolo, O., Massafra, R., Sciruicchio, V., Bellotti, R., 2003. Detection of subclinical brain electrical activity changes in Huntington's disease using artificial neural networks. Clin Neurophysiol. 114, 1237-1245. 10.1016/s1388-2457(03)00074-9 12842720

[ref-2077591383] Pokrovskaia, Z.A., Insarova, N.G., 1988. The EEG characteristics of patients with Huntington's chorea and their clinically healthy relatives. Zh Nevropatol Psikhiatr Im S S Korsakova. 88(3), 22-26. 2968029

[ref-4138590658] Scott, D.F., Heathfield, K.W., Toone, B., Margerison, J.H., 1972. The EEG in Huntington's chorea: a clinical and neuropathological study. J Neurol Neurosurg Psychiatry. 35, 97-102. 10.1136/jnnp.35.1.97 PMC493983 4260288

[ref-3268396446] Streletz, L.J., Reyes, P.F., Zalewska, M., Katz, L., Fariello, R.G., 1990. Computer analysis of EEG activity in dementia of the Alzheimer's type and Huntington's disease. Neurobiol Aging. 11, 15-20. 10.1016/0197-4580(90)90057-7 2139184

[ref-3792517424] van der Hiele, K., Jurgens, C.K., Vein, A.A., Reijntjes, R.H., Witjes-Ané, M.N., Roos, R.A., van Dijk, G., Middelkoop, H.A., 2007. Memory activation reveals abnormal EEG in preclinical Huntington's disease. Mov Disord. 22, 690-695. 10.1002/mds.21390 17266047

[ref-1707087785] Shoulson, I., Fahn, S., 1979. Huntington disease: clinical care and evaluation. Neurology. 29, 1-3. 10.1212/wnl.29.1.1 154626

[ref-4256579130] Folstein MF, Folstein SE, McHugh PR (1975). ""Mini-mental state". A practical method for grading the cognitive state of patients for the clinician". Journal of psychiatric research 12 (3): 189–98. 10.1016/0022-3956(75)90026-6 1202204

[ref-2836133875] Morgan, M.L., Cook, I.A., Rapkin, A.J., Leuchter, A.F., 2007. Neurophysiologic changes during estrogen augmentation in perimenopausal depression. Maturitas. 56, 54-60. 10.1016/j.maturitas.2006.05.010 16835012

[ref-1738402029] Cook, I.A., O'Hara, R., Uijtdehaage, S.H., Mandelkern, M., Leuchter, A.F., 1998b. Assessing the accuracy of topographic EEG mapping for determining local brain function. Electroencephalogr Clin Neurophysiol. 107, 408-414. 10.1016/s0013-4694(98)00092-3 9922086

[ref-1013477646] Langbehn, D.R., Brinkman, R.R., Falush, D., Paulsen, J.S., Hayden, M.R., 2004. A new model for prediction of the age of onset and penetrance for Huntington's disease based on CAG length. Clin Genet. 65, 267-277. 10.1111/j.1399-0004.2004.00241.x 15025718

[ref-2166046880] Cook, I.A., Leuchter, A.F., Uijtdehaage, S.H., Osato, S., Holschneider, D.H., Abrams, M., Rosenberg-Thompson, S., 1998a. Altered cerebral energy utilization in late life depression. J Affect Disord. 49(2), 89-99. 10.1016/s0165-0327(97)00192-4 9609672

[ref-1176912774] Penney, J.B., Vonsattel, J.P., MacDonald, M.E., Gusella, J.F., Myers, R.H., 1997. CAG repeat number governs the development rate of pathology in Huntington's disease. Ann Neurol. 41(5), 689-92. 10.1002/ana.410410521 9153534

[ref-1852447019] Leuchter, A.F., Newton, T.F., Cook, I.A., Walter, D.O., Rosenberg-Thompson, S., Lachenbruch, P.A., 1992. Changes in brain functional connectivity in Alzheimer-type and multi-infarct dementia. Brain. 115 ( Pt 5), 1543-1561. 10.1093/brain/115.5.1543 1422803

[ref-286052479] Browne, S.E., 2008. Mitochondria and Huntington's disease pathogenesis: insight from genetic and chemical models. Ann N Y Acad Sci. 147, 358-382. 10.1196/annals.1427.018 19076457

[ref-4231366531] Hasselbalch, S.G., Oberg, G., Sørensen, S.A., Andersen, A.R., Waldemar, G., Schmidt, J.F., Fenger, K., Paulson, O.B., 1992. Reduced regional cerebral blood flow in Huntington's disease studied by SPECT. J Neurol Neurosurg Psychiatry. 55, 1018-1023. 10.1136/jnnp.55.11.1018 PMC1015285 1469396

[ref-1264201509] Montoya, A., Price, B.H., Menear, M., Lepage, M., 2006. Brain imaging and cognitive dysfunctions in Huntington's disease. J Psychiatry Neurosci. 31, 21-29. PMC1325063 16496032

[ref-372215086] Marder, K., Zhao, H., Myers, R.H., Cudkowicz, M., Kayson, E., Kieburtz, K., Orme, C., Paulsen, J., Penney, J.B. Jr, Siemers, E., Shoulson, I., 2000. Rate of functional decline in Huntington's disease. Huntington Study Group. Neurology. 54, 452-458. 10.1212/wnl.54.2.452 10668713

[ref-3016332531] Brinkman, R. R., Mezei, M. M., Theilmann, J., Almqvist, E., and Hayden, M. R., 1997. The likelihood of being affected with Huntington disease by a particular age, for a specific CAG size. Am J Hum Genet 60, 1202-1210. PMC1712445 9150168

[ref-3656664016] Illarioshkin, S.N., Igarashi, S., Onodera, O., Markova, E.D., Nikolskaya, N.N., Tanaka, H., Chabrashwili, T.Z., Insarova, N.G., Endo, K., Ivanova-Smolenskaya, I.A., Tsuji, S., 1994. Trinucleotide repeat length and clinical progression in Huntington’s disease. Ann Neurol. 36, 630-635. 10.1002/ana.410360412 7944295

[ref-176083640] Stine, O. C., Pleasant, N., Franz, M. L., Abbott, M. H., Folstein, S. E., and Ross, C. A., 1993. Correlation between the onset age of Huntington's disease and length of the trinucleotide repeat in IT-15. Hum Mol Genet 2, 1547-1549. 10.1093/hmg/2.10.1547 8268907

[ref-2829138432] Paulsen, J. S., Wang, C., Duff, K., Barker, R., Nance, M., Beglinger, L., Moser, D., Williams, J. K., Simpson, S., Langbehn, D., and van Kammen, D. P., 2010. Challenges assessing clinical endpoints in early Huntington disease. Mov Disord. Jul 9. [Epub ahead of print]. 10.1002/mds.23337 PMC2978744 20623772

[ref-3220470817] Beglinger, L. J., O'Rourke, J.J., Wang, C., Langbehn, D.R., Duff, K., Paulsen, J.S.; Huntington Study Group Investigators, 2010. Earliest functional declines in Huntington disease. Psychiatry Res. 178(2), 414-8. 10.1016/j.psychres.2010.04.030 PMC3629818 20471695

